# Nach den Berufswahlpraktika ist vor der Wahl des Ausbildungsberufs: Vorhersage des Entscheids für die Weiterverfolgung eines Praktikumberufs im Berufswahlprozess

**DOI:** 10.1007/s11618-023-01174-8

**Published:** 2023-08-08

**Authors:** Jan Hofmann, Markus P. Neuenschwander

**Affiliations:** Zentrum Lernen und Sozialisation, Pädagogische Hochschule der Fachhochschule Nordwestschweiz, Bahnhofstrasse 6, 5210 Windisch, Schweiz

**Keywords:** Schnupperlehren, Praktika, Berufliche Grundbildung, Berufswahl, Lehrstellenwahl, Ausbildungsberuf, Trial apprenticeship, Vocational education and training, Career choice, Apprenticeship choice, Apprenticeship profession

## Abstract

In diesem Artikel wurde untersucht, welche Faktoren dazu führen, dass Jugendliche einen in ihren Schnupperlehren und Berufswahlpraktika ausprobierten Beruf (Praktikumberuf) in ihrem Berufswahlprozess als potenzieller Ausbildungsberuf weiterverfolgen. Als theoretische Grundlage für die Analysen diente Gottfredsons Theory of Circumscription, Compromise, and Self-Creation (TCCSC). Nach Geschlechter getrennt wurde eine Längsschnittstichprobe von 128 weiblichen und 202 männlichen Jugendlichen analysiert, die im siebten und neunten Schuljahr standardisierte Fragebögen ausfüllten und angaben, nach der obligatorischen Schulzeit eine berufliche Grundbildung zu beginnen. Die gerechneten Regressionsmodelle zeigten, dass das Lehrstellenangebot den Weiterverfolgungsentscheid für weibliche wie auch für männliche Jugendliche am stärksten beeinflusst (positiver Effekt). Ausschließlich bei den weiblichen Jugendlichen hat sich gezeigt, dass neben dem Lehrstellenangebot das Ausmaß führend-verkaufender Anforderungen des ausprobierten Praktikumberufs den Entscheid signifikant negativ beeinflusst. Die Ergebnisse stützen die in der TCCSC postulierte Zugänglichkeitsthese, wonach Personen Berufe mit größerer Wahrscheinlichkeit weiterverfolgen, wenn für diese Berufe eine gute Zugänglichkeit im Sinne eines großen Lehrstellenangebots besteht. Eine mögliche Erklärung für den negativen Einfluss führend-verkaufender Anforderungen auf den Weiterverfolgungsentscheid bei weiblichen Jugendlichen ist vor dem Hintergrund des in der TCCSC enthaltenen Kompatibilitätsgedankens, dass weibliche Jugendliche ihre führend-verkaufenden Fähigkeiten möglicherweise als zu gering einschätzen. Infolgedessen nehmen sie Berufe mit hohen führend-verkaufenden Anforderungen als für sich inkompatibel wahr und wenden sich von diesen Berufen ab. Diese Studie konnte mit der Untersuchung der Rolle von Praktikaberufen für den weiteren beruflichen Verlauf eine Lücke in der Berufswahlforschung angehen und bietet wichtige Anknüpfungspunkte für zukünftige Analysen.

## Abstract (en)

This article investigated the factors that lead adolescents to pursue a trial apprenticeship profession in their career choice process. Gottfredson’s Theory of Circumscription, Compromise, and Self-Creation (TCCSC) served as the theoretical basis for the analyses. A longitudinal sample of 128 female and 202 male adolescents who completed standardized questionnaires in seventh and ninth grades and who indicated that they would begin vocational education and training after compulsory schooling was analyzed separately by gender. Regression models showed that the number of apprenticeship positions has the strongest effect for both female and male adolescents (significant positive effect). Only for female adolescents, the extent of enterprising requirements of the trial apprenticeship profession was shown to have a negative effect on the decision to pursue a trial apprenticeship profession, controlling for the number of apprenticeship positions. The results support the accessibility hypothesis postulated in the TCCSC, according to which individuals are more likely to pursue professions they perceive as more accessible, i.e., with a large number of apprenticeship positions. In light of the compatibility idea contained in the TCCSC, a possible explanation for the negative effect of enterprising requirements of trial apprenticeship professions among female adolescents is that they may assess their enterprising skills as too low. As a result, they perceive professions with high enterprising requirements as incompatible and turn away from these professions. This study was able to address a gap in career choice research by examining the role of trial apprenticeship professions and offers important reference points for future research.

## Einleitung

In der Schweiz haben sich die meisten Jugendlichen mit ca. 16 Jahren für einen Ausbildungsberuf entschieden, den sie nach Abschluss der obligatorischen Schule in der beruflichen Grundbildung erlernen. Als Teil des Berufswahlprozesses absolviert das Gros der Jugendlichen ab einem Alter von rund 14 Jahren Schnupperlehren und Berufswahlpraktika (nachfolgend zusammengefasst als Praktika). Diesen Praktika kommt eine wichtige Funktion zu: Die „für einen bestimmten Bildungsgang erforderlichen oder nützlichen, außerhalb der Bildungseinrichtungen [absolvierten] praktischen Tätigkeiten“ (Duden o.J.) helfen, einen Beruf näher kennenzulernen und einen ersten authentischen Blick in die Berufswelt zu erhalten (Schweizerisches Dienstleistungszentrum Berufsbildung 2018). In den Praktika probieren die Jugendlichen meist verschiedene Berufe aus oder testen denselben Praktikumberuf in unterschiedlichen Betrieben. Nach dem Absolvieren der Praktika haben sie oft ein gutes Bild, ob ein Beruf für sie als Ausbildungsberuf in Frage kommt oder nicht. Aus der Forschung ist bekannt, dass das in den Praktika ausprobierte Berufsfeld häufig mit demjenigen Berufsfeld übereinstimmt, in welchem sich der später gewählte Ausbildungsberuf verorten lässt (Neuenschwander et al. 2018). Hinsichtlich beruflicher Dimensionen wie die Geschlechtstypik, der soziale Status oder berufliche Anforderungen z. B. im handwerklich-technischen Bereich hat sich gezeigt, dass zwischen Praktikumberuf und Ausbildungsberuf eine hohe Korrelation besteht (Hofmann und Neuenschwander 2021a, b, 2022). Auch wenn sich die Übereinstimmungs- und Korrelationswerte als hoch erwiesen, so waren sie statistisch jedoch nicht perfekt. Dies deutet darauf hin, dass sich ein Teil der Jugendlichen nach dem Absolvieren der Praktika gegen eine Weiterverfolgung des Praktikumberufs im eigenen Berufswahlprozess entscheidet und einen anderen Beruf für ihre berufliche Grundbildung wählt als den zuvor in den Praktika ausprobierten. Unter welchen Bedingungen Jugendliche von einem Praktikumberuf abkehren, wurde bislang nicht untersucht. Kenntnisse über diese Bedingungen zu erhalten ist wichtig, da Jugendliche in den Praktika häufig ihre gehegten Wunschberufe bzw. angestrebten Berufsfelder ausprobieren. So zeigten Neuenschwander et al. (2018), dass das Berufsfeld des Wunschberufs mit dem Berufsfeld des Praktikumberufs zu 56 % übereinstimmt. Um Jugendliche in ihren individuellen beruflichen Laufbahnen zu unterstützen, braucht es Wissen über allfällige Faktoren, welche die Jugendlichen an der Umsetzung ihrer beruflichen Wünsche hindern.

Die Determinanten eines Berufswechsels zwischen dem Absolvieren von Praktika und der Wahl eines Ausbildungsberufs wurden anhand der folgenden Forschungsfrage untersucht: Unter welchen Bedingungen entscheiden sich Jugendliche nach dem Absolvieren von Praktika, von einem erprobten Praktikumberuf im eigenen Berufswahlprozess abzukehren bzw. diesen Beruf als potenzieller Ausbildungsberuf weiterzuverfolgen?

Zur Beantwortung der Forschungsfrage wurde auf die Theory of Circumscription, Compromise, and Self-Creation (TCCSC) von Gottfredson (2002) zurückgegriffen. Diese Theorie behandelt Abwäge- und Anpassungsprozesse bei der Berufswahl und eignet sich daher als theoretische Grundlage für diese Studie. Die TCCSC wurde bislang vor allem in Studien in der frühen und späten Kindheit angewendet und kam nur vereinzelt bei Jugendlichen und Erwachsenen zum Einsatz (Gottfredson 2002). Die vorliegende Studie zu Anpassungsprozessen in der Berufswahl von Jugendlichen stärkt somit den Forschungsstand zu einer eher wenig erforschten Zielgruppe (Ratschinski 2009; Steinritz et al. 2016). Außerdem kann auch aus forschungsmethodischer Hinsicht ein wichtiger Beitrag geleistet werden. Bisherige Studien, welche die TCCSC als theoretische Grundlage verwendeten, nutzten häufig ein experimentelles Design wie beispielsweise den choice dilemma approach. Dem Verfahren wird eine gewisse Künstlichkeit angelastet (Leung und Plake 1990), da die Studienteilnehmenden aus verschiedenen Berufspaaren jeweils einen favorisierten Beruf wählen. Der vorliegende Beitrag kann diese Limitation überwinden, da nicht bloß geäußerte berufliche Präferenzen untersucht werden, sondern konkrete, in der Arbeitswelt ausgeübte Berufswahlhandlungen. Weiter wurde die Zugänglichkeit von Berufen im Sinne verfügbarer Stellenangebote in bisherigen Forschungsarbeiten kaum berücksichtigt, obwohl dieser Aspekt gemäß TCCSC bei der Berufswahl entscheidungsrelevant ist. Mit dem Einbezug des Lehrstellenangebots als Zugänglichkeitsaspekt von Berufen kann diese Studie auch in diesem Bereich neue Erkenntnisse liefern.

### Gottfredsons Theory of Circumscription, Compromise and Self-Creation (TCCSC)

#### Selbstkonzept und Vorstellungen zu Berufen

Ein wichtiger Faktor, der die Berufswahl beeinflusst, ist gemäß Gottfredsons (2002) TCCSC das Selbstkonzept, also die kognitive Vorstellung darüber, wer man ist. Personen basieren ihre Handlungen auf ihrem Selbstkonzept und versuchen, es in seinem Grundgerüst zu wahren. Handlungen werden entsprechend so interpretiert, dass sie mit dem Selbstkonzept kompatibel sind (Gottfredson 2002).

Gemäß der TCCSC machen sich Menschen nicht nur ein Bild darüber, wer sie sind, sondern sie haben auch individuelle Vorstellungen zu Berufen (engl. images of occupation). Sie stellen sich die mit der Ausübung eines Berufs verbundenen Arbeiten vor und schätzen die Arbeitsbedingungen und die Anforderungen ein, welche ein Beruf mit sich bringt. Sie haben eine kognitive Repräsentation, welche Persönlichkeiten diejenigen Menschen haben, welche in einem bestimmten Beruf arbeiten, stellen sich deren Leben vor und beurteilen die Angemessenheit der Berufsausübung für bestimmte Personengruppen und sich selbst (Gottfredson 2002). Bei der Berufswahl vergleichen sie hierzu ihr Selbstkonzept und ihre individuellen Vorstellungen zu Berufen und unterziehen sich einer Kompatibilitätsprüfung.

#### Kompatibilität zwischen Selbstkonzept und beruflichen Dimensionen

Unter Kompatibilität wird die Einschätzung einer Person verstanden, wie gut ein Beruf gemäß ihrer Vorstellung zu ihrem Selbstkonzept passt (Gottfredson 2002). Je höher die Kompatibilität zwischen Selbstkonzept und Beruf eingeschätzt wird, desto grösser ist die Präferenz, diesen Beruf zu wählen und später auszuüben (Gottfredson 2002). Während karrierebezogene Überlegungen bis zum neunten Lebensjahr noch hauptsächlich auf Kompatibilitätseinschätzungen bezüglich Geschlecht und Geschlechtstypik eines Berufs basieren und zwischen dem neunten und 14. Lebensjahr der mit einem Beruf verbundene soziale Status im Vordergrund steht, liegt der Fokus ab dem 14. Lebensjahr auf Kompatibilitätsbestrebungen zwischen den beruflichen Interessen und den mit einem Beruf verbundenen beruflichen Anforderungen. Was ein Beruf an Aufgaben und Anforderungen bietet, ist in dieser Phase des Berufswahlprozesses die entscheidungsrelevante berufliche Dimension (Gottfredson 2002). Jugendliche achten vor allem darauf, dass sie im Beruf ihre präferierten Aktivitäten ausüben und ihre beruflichen Interessen ausleben können. Eine hohe Person-Beruf-Kompatibilität zwischen den beruflichen Interessen und den mit einem Beruf verbundenen beruflichen Anforderungen wird angestrebt. Je grösser die Kompatibilität zwischen den beruflichen Interessen einer Person und dem, was ein bestimmter Beruf an Anforderungen bietet, ist bzw. wahrgenommen wird, desto grösser ist die Wahrscheinlichkeit eines Entscheids für die Weiterverfolgung jenes Berufs im eigenen Berufswahlprozess (Gottfredson 2002). In der Vergangenheit stützten mehrere Studien die entscheidungsrelevante Rolle von beruflichen Interessen im Berufswahlprozess (Blanchard und Lichtenberg 2003; Hesketh et al. 1990; Ratschinski 2009; Wee 2014).

#### Zugänglichkeit, Eingrenzung und Kompromiss

Die Wahl eines Berufs hängt neben der Kompatibilität zwischen Person und Beruf vor allem von der (wahrgenommenen) Zugänglichkeit (engl. accessibility) des Berufs ab (Gottfredson 2002). Ein Beruf kann einerseits von einer Person subjektiv als schwer zugänglich wahrgenommen werden; andererseits besteht für gewisse Berufe de facto ein begrenztes Stellenangebot im Arbeitsmarkt (Gottfredson 2002). Personen verfolgen in ihrem Berufswahlprozess deshalb häufig nicht bloß einen Beruf, sondern entwickeln für sich einen Bereich akzeptabler Alternativen (engl. zone of acceptable alternatives). Dieser Bereich repräsentiert die Reichweite an Berufen, welche eine Person für sich als akzeptabel erachtet und für welche eine gewisse Präferenz besteht. Im Laufe des Berufswahlprozesses wird der Bereich akzeptabler Berufe infolge wahrgenommener Kompatibilitätsunstimmigkeiten und/oder (wahrgenommener) Zugänglichkeitsschwierigkeiten fortlaufend eingeengt, indem unpassende Berufe ausgeschlossen werden. Dieser Prozess wird als Eingrenzung (engl. circumscription) bezeichnet (Gottfredson 2002). Bei der Eingrenzung hat die Zugänglichkeit eines Berufs eine dominierende Rolle: Bevorzugte Berufe werden für weniger kompatible, jedoch als zugänglicher wahrgenommene Berufe aufgegeben (Gottfredson 2002). Dieser Prozess wird mit dem Begriff des Kompromisses (engl. compromise) zusammengefasst. Zugänglichkeitsschwierigkeiten können sich zum Beispiel durch fehlende Arbeitsstellen in einem bestimmten Beruf (Zugänglichkeit als berufliche Dimension), negative Einstellungspraktiken (z. B. Diskriminierung) oder mit familiären Verpflichtungen verbundenen Aspekten (Angebotsmangel an Teilzeitstellen) zeigen (Gottfredson 2002).

Im Kontext der Wahl von Praktikaberufen und darauffolgender Entscheidungen für bzw. gegen die Weiterverfolgung der in den Praktika ausprobierten Berufe ist das Absolvieren von Praktika bei den meisten Jugendlichen Teil des Eingrenzungsprozesses. Einerseits können beim Austesten von Berufen Kompatibilitätsunstimmigkeiten festgestellt werden. Andererseits kann es zu Zugänglichkeitsschwierigkeiten bei der Wahl der Ausbildungsberufe kommen, was die Jugendlichen zum Vollzug von Kompromissen zwingen kann. Im Gegensatz zu den Berufen, welche Jugendliche in ihren beruflichen Grundbildungen erlernen, beschränkt sich die Auswahl an Berufen, die in Praktika ausprobiert werden können, nicht auf die Berufe des Lehrstellenmarktes, sondern erstreckt sich auf eine breitere Palette an Berufen aus dem Gesamtarbeitsmarkt. Bei der Wahl eines Ausbildungsberufs schränkt sich die Auswahl an Berufen entsprechend ein und die Jugendlichen müssen sich dem Lehrstellenangebot des Arbeitsmarkts anpassen (Stuhlmann 2009). Hirschi (2009) zeigte für den Schweizer Lehrstellenmarkt, dass für gewisse berufliche Anforderungen bzw. Berufsbereiche kaum Lehrstellen angeboten werden. Lehrstellen in den Holland (1997) Berufsbereichen *untersuchend-forschend, künstlerisch-kreativ* und *führend-verkaufend* werden mit lediglich 1 %, 3 % und 5 % aller verfügbaren Lehrstellen nur schwach abgedeckt. Auf der anderen Seite gibt es relativ viele Lehrstellen in den Berufsbereichen *handwerklich-technisch* (54 %), *ordnend-verwaltend* (27 %) und *erziehend-pflegend* (11 %).

### Umsetzung in dieser Studie

#### Hypothesen

Eingebettet in die lokalen wirtschaftsstrukturellen Bedingungen nutzen Jugendliche Praktika häufig dazu, um gehegte Wunschberufe und angestrebte Berufsfelder zu erproben (Neuenschwander et al. 2018). Bei den meisten kommt es in der Folge zum Prozess der Eingrenzung nach Gottfredson (2002), wobei eine didaktische Begleitung durch Lehrpersonen und Praktikumsverantwortliche maßgeblich Anteil daran hat, dass dieser Eingrenzungsprozess für die Jugendlichen erfolgreich verläuft (Bergzog 2011; Driesel-Lange et al. 2013). Auf Basis der TCCSC ist davon auszugehen, dass die Zugänglichkeit eines Berufs die Eingrenzung am stärksten bestimmt, d. h. bevorzugte Berufe werden für weniger kompatible, jedoch als zugänglicher wahrgenommene Berufe aufgegeben (Kompromiss). Neben dem Zugänglichkeitsaspekt ist mit Bezug auf das Alter der Jugendlichen beim Absolvieren der Praktika außerdem anzunehmen, dass die Person-Beruf-Kompatibilität zwischen den beruflichen Interessen der Jugendlichen und den beruflichen Anforderungen der ausprobierten Praktikaberufe den Weiterverfolgungsentscheid ebenfalls beeinflusst. Um die Forschungsfrage nach den Bedingungen des Entscheids für die Weiterverfolgung eines Praktikumberufs im Berufswahlprozess zu beantworten, wurden folgende Hypothesen zur Überprüfung aufgestellt:

##### Hypothese 1

Mit zunehmender Anzahl angebotener Lehrstellen zu einem von den Jugendlichen ausprobierten Praktikumsberuf erhöht sich die Wahrscheinlichkeit eines Entscheids für die Weiterverfolgung dieses ausprobierten Berufs im Berufswahlprozess.

##### Hypothese 2

Je grösser die Person-Beruf-Kompatibilität zwischen den beruflichen Interessen der Jugendlichen und den beruflichen Anforderungen eines ausprobierten Praktikumberufs ist, desto grösser ist die Wahrscheinlichkeit eines Entscheids für die Weiterverfolgung dieses Berufs im Berufswahlprozess.

#### Geschlechtermoderation

Gemäß Gottfredson (2002) bestehen zwischen weiblichen und männlichen Personen Unterschiede bei der Priorisierung beruflicher Dimensionen für die Berufswahl. Eine Studie von Taylor und Pryor (1985) gibt Hinweise darauf, dass es Unterschiede zwischen weiblichen und männlichen Personen hinsichtlich des prädiktiven Einflusses beruflicher Interessen auf Berufsentscheide gibt. Die Autoren zeigten, dass Frauen karrierebezogene Entscheide eher auf Basis ihrer Interessen treffen, während Männer bereit sind, bei Entscheidungen Interessenskonformität für andere Aspekte wie hohes Prestige und eine geschlechtstypische Wahl aufzugeben. Es ist möglich, dass der Einfluss der Person-Beruf-Kompatibilität zwischen den beruflichen Interessen der Jugendlichen und den beruflichen Anforderungen der von ihnen ausprobierten Praktikaberufen auf den Weiterverfolgungsentscheid durch das Geschlecht moderiert wird. Aus diesem Grund wurde eine potenzielle Geschlechtermoderation in der vorliegenden Studie überprüft.

## Methode

### Stichprobe und Erhebungsmethodik

Die Forschungsfrage wurde mit Daten aus dem Schweizer Längsschnittprojekt „WiSel – Wirkungen der Selektion“ (Neuenschwander et al. 2016) untersucht, in welchem Jugendliche aus den Deutschschweizer Kantonen Aargau, Basel-Landschaft, Bern und Luzern Fragen zu ihrem Berufswahlprozess beantworteten. Das WiSel-Projekt startete 2011, als die Jugendlichen im fünften Schuljahr waren, und wurde bis zum Ende der obligatorischen Schulzeit mit drei weiteren Erhebungen in den Jahren 2012, 2013 und 2016 fortgeführt. Bei der ersten Erhebung wurde eine zufällige Auswahl an Schulen der vier Kantone mit Klassen des fünften Schuljahrs für eine Studienteilnahme angeschrieben. In den darauffolgenden Erhebungen wurden dieselben Jugendlichen erneut für eine Studienteilnahme angefragt. Falls Jugendliche in eine Klasse wechselten, die noch nicht an der Studie teilgenommen hatte, wurden die Schülerinnen und Schüler dieser Klasse ebenfalls für eine Studienteilnahme angefragt. Die Fragestellung wurde mit Daten aus dem WiSel-Projekt untersucht, da im Projekt neben den Ausbildungsberufen auch die Berufe der absolvierten Praktika und in früheren Erhebungswellen mögliche Determinanten für die Wahl der Praktika erhoben wurden. Neben Angaben aus dem WiSel-Projekt wurden für die Operationalisierung des Lehrstellenangebots Daten des Nahtstellenbarometers der Jahre 2018 bis 2020 verwendet (Golder und Mousson 2019, 2020, 2021). Das Nahtstellenbarometer erhebt als einziges Projekt in der Schweiz die Angebotsseite an Lehrstellen aus Sicht der Unternehmen in repräsentativen Umfragen.

Zur Prüfung der formulierten Hypothesen wurden aus dem WiSel-Projekt die Daten der 2013er- und 2016er-Erhebung verwendet, als die Jugendlichen im siebten bzw. neunten Schuljahr waren. Im neunten Schuljahr haben die meisten Jugendlichen ihre Praktika bereits absolviert und einen Ausbildungsberuf gewählt (d. h. der Berufswahlprozess ist abgeschlossen). Weil das Absolvieren der Praktika im neunten Schuljahr noch nicht allzu lange zurückliegt, ist anzunehmen, dass Jugendliche zuverlässig und ohne größere Erinnerungsverzerrungen über ihre Praktika-Erfahrungen berichten können. Im siebten Schuljahr haben die meisten Jugendlichen hingegen noch keine Praktika absolviert. Sie stehen am Anfang ihres Berufswahlprozesses. An der ersten dieser beiden Erhebungen (T_1_) nahmen 1515 Jugendliche teil (weiblich: *n* = 732, männlich: *n* *=* 783). Von diesen Jugendlichen füllten 698 auch den Fragebogen an der zweiten Erhebung (T_2_) aus (weiblich: *n* = 356, männlich: *n* = 342), was einer Rücklaufquote von 46 % (weiblich: 49 %, männlich: 44 %) entspricht. Insgesamt hatten 2376 Jugendliche zu T_2_ teilgenommen (weiblich: *n* = 1258, männlich: *n* = 1105; 13 Jugendliche ohne Geschlechtsangabe). Mit Blick auf die Forschungsfrage wurde die Studienstichprobe auf jene Jugendlichen eingeschränkt, welche an beiden Erhebungen zu T_1_ und T_2_ teilgenommen hatten, mindestens eine Schnupperlehre oder ein Praktikum absolvierten und angaben, nach der obligatorischen Schulzeit eine berufliche Grundbildung zu starten (*N* = 330, weiblich: *n* = 128, männlich: *n* = 202). Das Durchschnittsalter der Studienstichprobe lag zu T_1_ bei 13,3 Jahren (*SD* = 0,6 Jahre). T_2_ wurde rund 28 Monate nach T_1_ durchgeführt, als die Jugendlichen im Durchschnitt 15,6 Jahre alt waren.

Allfällige Selektionseffekte der Längsschnittstichprobe wurden für alle T_2_-Rohvariablen mittels *t*-Tests (für die metrischen Variablen) und Mann-Whitney-U-Tests (für die ordinalskalierten Variablen) getrennt nach Geschlechtergruppen in SPSS 25.0 geprüft. Im Vergleich zwischen dem verwendeten Längsschnittsample und Jugendlichen, welche die obigen Kriterien ebenfalls erfüllten, jedoch nur zu T_2_ an der Studie teilnahmen, zeigten sich keine Selektionseffekte oder nur mit einer geringen bis mittleren Effektstärke. Signifikante Unterschiede ergaben sich bei den weiblichen Jugendlichen für folgende Variablen: Lehrstellenangebot des zweiten Praktikumberufs, *t*(215,19) = −2,66, *p* = 0,008, |*d*| = 0,26; handwerklich-technische Anforderungen des vierten Praktikumberufs, *t*(154,28) = −3,48, *p* < 0,001, |*d*| = 0,40; untersuchend-forschende Anforderungen des zweiten Praktikumberufs, *t*(489) = 2,99, *p* = 0,003, |*d*| = 0,32 und des dritten Praktikumberufs, *t*(445) = 3,23, *p* < 0,001, |*d*| = 0,36; führend-verkaufende Anforderungen des vierten Praktikumberufs, *t*(367) = 2,28, *p* = 0,023, |*d*| = 0,28 und ordnend-verwaltende Anforderungen des vierten Praktikumberufs, *t*(151,16) = 3,01, *p* = 0,003, |*d*| = 0,35. Bei den männlichen Jugendlichen zeigten sich signifikante Unterschiede in den folgenden Variablen: untersuchend-forschende Anforderungen des zweiten Praktikumberufs, *t*(591) = 1,97, *p* = 0,049, |*d*| = 0,18 und des dritten Praktikumberufs, *t*(507) = 2,67, *p* = 0,008, |*d*| = 0,26. Aufgrund der geringen Effektstärken sind die Selektionseffekte als unbedenklich einzustufen und relevante Verzerrungen bei den Ergebnissen sind nicht zu erwarten.

### Variablen

Nachfolgend wird zuerst die Erhebung der Praktikaberufe geschildert, gefolgt von einer Beschreibung der in dieser Studie verwendeten Konzepte.

#### Erhebung der Praktikaberufe

Die Jugendlichen gaben im neunten Schuljahr (T_2_) an, in welchen Berufen sie Schnupperlehren und Praktika absolviert hatten. Mit einem offenen Antwortformat wurden sie gebeten, die folgende Frage zu beantworten: „In welchen Berufen […] hast du die verschiedenen Schnupperlehren gemacht?“ (analoge Formulierung für Praktika). Die Jugendlichen konnten maximal vier Schnupperlehr- und vier Praktikaberufe angeben. Insgesamt wurden 1124 Schnupperlehrberufe (weiblich: 445; männlich: 679) und 26 Praktikaberufe (weiblich: 14; männlich: 12) genannt. Im Durchschnitt probierten die Jugendlichen vier Schnupperlehrberufe und < 1 Praktikumberuf aus (weibliche Jugendliche: 4 Schnupperlehrberufe, < 1 Praktikumberuf; männliche Jugendlichen: 4 Schnupperlehrberufe, < 1 Praktikumberuf). Falls die Jugendlichen mehr als vier Schnupperlehren bzw. mehr als vier Praktika absolviert hatten, waren diejenigen Schnupperlehren/Praktika zu nennen, die für die Berufswahl am relevantesten waren. Die Berufe wurden zu Berufsgattungen gemäß der International Standard Classification of Occupations zusammengefasst (ISCO-08; International Labour Organization ILO 2012), auf deren Basis die verschiedenen Berufskodierungen (Lehrstellenangebot, berufliche Anforderungen) durchgeführt wurden.

#### Geschlecht

Im siebten (T_1_) und im neunten (T_2_) Schuljahr wurden die Jugendlichen über folgende Aufforderung gebeten, ihr Geschlecht anzugeben: „Bitte gib dein Geschlecht an“. Zur Auswahl standen die beiden Antwortmöglichkeiten „weiblich“ und „männlich“. Um fehlende Werte zu minimieren, wurden die Angaben aus den beiden Erhebungszeitpunkten kombiniert. Es gab keine Veränderungen in den Geschlechtsangaben zwischen den beiden Messzeitpunkten.

#### Entscheid für Weiterverfolgung eines Praktikumberufs

Im neunten Schuljahr (T_2_) schätzten die Jugendlichen zu jedem ihrer genannten Schnupperlehr- und Praktikaberufe ein, ob das Absolvieren des Schnupperlehr- bzw. Praktikumberufs dazu geführt hatte, dass sie sich für bzw. gegen die Weiterverfolgung dieses Berufs im eigenen Berufswahlprozess entschieden hatten. Hierzu wurden sie gebeten, die folgende Frage zu beantworten: „Hat eine der Schnupperlehren dazu geführt, dass du dich für/gegen diesen Beruf entschieden hast?“ (analoge Formulierung für Praktika). Folgende Optionen standen zur Beantwortung der Frage zur Verfügung: 1 (*dafür*), 2 (*dagegen*), 3 (*weder noch*). Für diese Studie wurden die Werte wie folgt umkodiert: 1 (*dagegen*), 2 (*weder noch*), 3 (*dafür*). Zu den angegebenen 1150 Schnupperlehr- bzw. Praktikaberufen wurde 241-mal mit „dagegen“ (weiblich: 107 [23 % aller Antworten weiblicher Jugendlicher], männlich: 134 [19 % aller Antworten männlicher Jugendlicher]), 241-mal mit „unentschlossen“ (weiblich: 86 [19 %], männlich: 155 [22 %]) und 668-mal mit „dafür“ (weiblich: 266 [58 %], männlich: 402 [58 %]) geantwortet.

#### Lehrstellenangebot zu Praktikaberufen

Das Lehrstellenangebot zu den von den Jugendlichen angegebenen Schnupperlehr- und Praktikaberufen wurde mit Daten des Nahtstellenbarometers der Jahre 2018 bis 2020 erfasst und über die Variable *Lehrstellenangebot* abgebildet (Golder und Mousson 2019, 2020, 2021). Verwendet wurden die Daten aus Unternehmensfragebögen der zweiten der beiden jährlich durchgeführten Erhebungswellen (Erhebungszeit: Juli bis September). Die Fragebögen wurden von Personen aus ausbildenden Schweizer Unternehmen mit mindestens zwei Angestellten auf Papier oder online ausgefüllt. Die Unternehmen, die an der Umfrage teilnahmen, stammten aus dem Betriebs- und Unternehmensregister des Bundesamts für Statistik und wurden nach dem Zufallsprinzip ausgewählt. Im Datensatz des Nahtstellenbarometers lag für die Daten eine Gewichtung nach Sprachregion, Betriebsgröße und Wirtschaftszweig (NOGA-Verteilung) vor. Diese Gewichtung wurde bei der Erstellung der Variable zum Lehrstellenangebot berücksichtigt. Zur Beantwortung der Fragestellung in dieser Studie wurden Daten genutzt, die wie folgt erhoben wurden: Über die Aufforderung „Beantworten Sie die folgenden Fragen bitte für alle von Ihrem gesamten Unternehmen angebotenen beruflichen Grundbildungen, indem Sie jeweils die entsprechenden Kolonnen für jeden Beruf einzeln ausfüllen“ wurden die Unternehmen gebeten, alle Berufe anzugeben, in welchen in ihrem Unternehmen eine berufliche Grundbildung absolviert werden konnte. Über die Frage „Wie viele Lehrstellen hat Ihr Unternehmen [Jahr] nun definitiv neu vergeben? Damit sind nur Lehrstellen gemeint, die [Jahr] im 1. Lehrjahr neu beginnen“ sollten die Unternehmen angeben, wie viele Lehrstellen ihr Unternehmen in einem bestimmten Jahr definitiv neu vergeben hatte. Ein offenes Antwortformat stand jeweils zur Verfügung, wobei im Nahtstellenbarometer-Datensatz bis zu vier (2018) bzw. sieben (2019, 2020) Ausbildungsberufe berücksichtigt wurden. Die pro Beruf angegebenen Lehrstellen wurden für jeden der drei Jahres-Datensätze z‑standardisiert, um große Unterschiede in der Gesamtzahl angebotener Lehrstellen zwischen den Jahren abzuschwächen. Danach wurden aus den z‑standardisierten Werten die Mittelwerte zur Anzahl Lehrstellen pro Ausbildungsberuf über die drei Jahre hinweg berechnet. Die Lehrstellenangebote zu den Berufen wurden auf Ebene der Berufsgattungen gemäß der International Standard Classification of Occupations zusammengefasst (ISCO-08), indem aus den zu einer Berufsgattung zugehörigen Berufen bzw. deren Lehrstellenangeboten der Mittelwert berechnet wurde. Den nach ISCO-08 kodierten Praktikaberufen wurde anschließend der jeweilige Lehrstellenangebotswert zugewiesen. Je höher der Wert in der Variable ist, desto grösser ist das Lehrstellenangebot zu den ausprobierten Praktikaberufen.

#### Person-Beruf-Kompatibilität berufliche Interessen/Anforderungen zu Praktikaberufen

Die Person-Beruf-Kompatibilität zwischen den beruflichen Interessen der Jugendlichen und den beruflichen Anforderungen der von den Jugendlichen ausprobierten Praktikaberufe wurde über Angaben der Jugendlichen zu ihren beruflichen Interessen sowie Daten zu beruflichen Anforderungen von Berufen gebildet. Sowohl die beruflichen Interessen als auch die beruflichen Anforderungen wurden in Anlehnung an Hollands (1997) RIASEC-Berufsklassifizierungssystem entlang der folgenden sechs Interessens- bzw. Umweltbereiche operationalisiert: handwerklich-technisch, untersuchend-forschend, künstlerisch-kreativ, erziehend-pflegend, führend-verkaufend und ordnend-verwaltend.

Die beruflichen Interessen der Jugendlichen wurden im siebten Schuljahr (T_1_) erhoben. Die Jugendlichen wurden um ihre Einschätzungen gebeten, wie gerne sie bestimmte Tätigkeiten ausführten: „Bitte schätze ein, wie gerne du folgende Tätigkeiten ausführst“. Die einzuschätzenden Tätigkeiten waren an das Berufsregister des Explorix von Jörin et al. (2004) angelehnt und lauteten: „Handwerkliche und technische Tätigkeiten (montieren, reparieren, anfertigen)“, „forschende und untersuchende Tätigkeiten (entwickeln, erfinden, verstehen)“, „künstlerische und kreative Tätigkeiten (tanzen, musizieren, schreiben, gestalten)“, „erziehende und pflegende Tätigkeiten (erklären, beraten, umsorgen)“, „verkaufende und führende Tätigkeiten (überzeugen, verhandeln, leiten)“ und „ordnende und verwaltende Tätigkeiten (sammeln, organisieren, überprüfen)“. Die Einschätzungen wurden auf einer Skala von 1 (*sehr ungerne*) bis 6 (*sehr gerne*) erfasst.

Die Operationalisierung der beruflichen Anforderungen erfolgte über Schmidts (2008) Klassifizierung von Berufen nach beruflichen Anforderungen (Hofmann und Neuenschwander 2022). Für Schmidts Klassifizierung von Berufen schätzten 21 Berufsberaterinnen und Berufsberater die im Jahr 2006 in der Schweiz am häufigsten gewählten Ausbildungsberufe hinsichtlich ihrer Anforderungen in den sechs Interessens- bzw. Umweltbereichen von Holland (1997) ein. Jeder Ausbildungsberuf wurde von mindestens neun und maximal 12 Berufsberaterinnen und Berufsberatern eingeschätzt. Die Skala für die Einschätzungen reichte von 1 (*äußerst tief*) bis 6 (*äußerst hoch*). Die Anforderungseinschätzungen der Berufsberaterinnen und Berufsberater wurden pro Ausbildungsberuf und für jeden der sechs Interessens- bzw. Umweltbereiche zu Mittelwerten verrechnet. Für die vorliegende Studie wurden die Angaben zu den beruflichen Anforderungen anschließend pro (Ausbildungs‑)Beruf auf Berufsgattungsebene gemäß ISCO-08 (ILO 2012) zusammengefasst, indem aus den Angaben zu den Berufen derselben Berufsgattung der Mittelwert berechnet wurde. Dieses Vorgehen minimierte fehlende Werte in den Variablen zu beruflichen Anforderungen.

Die Person-Beruf-Kompatibilitätsvariablen wurden erstellt, indem pro Holland-Interessens‑/Umweltbereich der z‑standardisierte Interessenswert vom z‑standardisierten Anforderungswert subtrahiert wurde. Die Konstrukte wurden z‑standardisiert verrechnet, um allfällige Unterschiede in den Anwortspannweiten zwischen den Konstrukten zu berücksichtigen.

Die Person-Beruf-Kompatibilität zwischen den beruflichen Interessen der Jugendlichen und den beruflichen Anforderungen der von ihnen ausprobierten Praktikaberufe wurde mit einer Variable pro Holland-Interessens‑/Umweltbereich in die Analysen aufgenommen. Für jede der sechs Person-Beruf-Kompatibilitätsvariablen gilt: Je höher der Wert ist, desto mehr übersteigen die beruflichen Anforderungen des Praktikumberufs die beruflichen Interessen der Jugendlichen.

### Auswertungsmethodik

Um die Forschungsfrage zu beantworten und die Hypothesen zu überprüfen, wurde ein ordinal-logistisches Regressionsmodell in Mplus 8.1 gerechnet. Der Entscheid für die Weiterverfolgung eines Praktikumberufs wurde als ordinalskalierte abhängige Variable spezifiziert. Als Prädiktoren dienten das Lehrstellenangebot und die sechs Variablen zu Person-Beruf-Kompatibilität zwischen den beruflichen Interessen und den beruflichen Anforderungen. Verwendet wurde ein Datensatz im Längsformat, in welchem die von den Jugendlichen ausprobierten Schnupperlehr- und Praktikaberufe bzw. die daraus kodierten Variablen (Weiterverfolgungsentscheid, Lehrstellenangebot, Person-Beruf-Kompatibilität berufliche Interessen/Anforderungen) genestet in den Jugendlichen vorlagen. In Mplus wurde die Nestung der Daten über *type=complex* berücksichtigt.

Für die Berechnung des Regressionsmodells wurde die standardmäßig zum Einsatz kommende probit-Link Funktion mit Delta-Parametrisierung und der *weighted least square mean and variance adjusted (WLSMV)*-Schätzer genutzt. Fehlende Werte wurden aufgrund des komplexen Studiendesigns (Längsschnittstichprobe, variable Anzahl an absolvierten Praktika pro Person, ordinalskalierte abhängige Variable) nicht geschätzt/imputiert (listwise deletion).

Um die Geschlechtermoderationseffekte zu testen, wurde das Regressionsmodell mit Gruppenvergleich nach Geschlecht berechnet. Geschlechtergruppenunterschiede wurden mit χ^2^-Differenztests geprüft. Hierzu wurden für jeden Effekt separat zwei Modelle miteinander verglichen: In einem Modell waren alle Effekte bis auf ein Effekt zwischen den Geschlechtern gleichgesetzt, im anderen Modell waren alle Effekte zwischen den Geschlechtern gleichgesetzt. Signifikante χ^2^-Differenzwerte wurden als Indikator für signifikante Unterschiede zwischen weiblichen und männlichen Jugendlichen in den geprüften Effekten verwendet.

In einem post-hoc Verfahren wurde außerdem überprüft, in welchem Ausmaß die beruflichen Anforderungen der von den Jugendlichen ausprobierten Praktikaberufe unabhängig von Kompatibilitätsbezügen zu den beruflichen Interessen einen Einfluss auf den Weiterverfolgungsentscheid haben. Mit dieser Überprüfung kann die potenzielle Fehlannahme einer Abhängigkeit des Weiterverfolgungsentscheids von individuellen beruflichen Interessen als Teil des Selbstkonzepts vorgebeugt werden. Aus Multikollinearitätsgründen wurden nur jene beruflichen Anforderungen geprüft, für welche unter Berücksichtigung von Kompatibilitätsbezügen zu den beruflichen Interessen signifikante Effekte vorgefunden wurden. Für die Überprüfung wurden dieselben beruflichen Anforderungen genutzt, welche bei der Erstellung der Person-Beruf-Kompatibilitätsvariablen zum Einsatz kamen. Alle berichteten Ergebnisse basieren auf einem zweiseitigen Signifikanzniveau.

## Ergebnisse

### Hypothesenprüfung

In Tab. 1 sind die Ergebnisse des gerechneten Regressionsmodells zur Vorhersage des Entscheids für die Weiterverfolgung eines Praktikumberufs dargestellt.

**Table 1:** 

	Weibliche Jugendliche (*n* = 123)	Männliche Jugendliche (*n* = 188)
Lehrstellenangebot	0,32***	0,17**
P-B‑K handw.-techn. I./A	0,07	0,01
P-B‑K unters.-forsch. I./A	0,02	0,03
P-B‑K künstl.-kreat. I./A	−0,14	0,01
P-B‑K erzieh.-pfleg. I./A	−0,04	−0,01
P-B‑K führ.-verkauf. I./A	−0,17*	**0,08**
P-B‑K ordn.-verw. I./A	−0,06	−0,03
*R*^*2*^	0,13**	0,03

Anmerkungen: standardisierte Koeffizienten β

*P-B‑K* Person-Beruf-Kompatibilität; *I./A.* Interessen/Anforderungen, *handw.-techn.* handwerklich-technische, *unters.-forsch.* untersuchend-forschende, *künstl.-kreat.* künstlerisch-kreative, *erzieh.-pfleg.* erziehend-pflegende, *führ.-verkauf.* führend-verkaufende, *ordn.-verw.* ordnend-verwaltende

fett markierte Werte verweisen auf signifikante Geschlechterunterschiede in Koeffizienten

Kodierung der ordinalskalierten abhängigen Variable Entscheid für die Weiterverfolgung eines Praktikumberufs: 1 = dagegen, 2 = unentschlossen, 3 = dafür; Prädiktoren sind metrisch skaliert

*p < 0,05 (zweiseitig), **p < 0,01 (zweiseitig), ***p < 0,001 (zweiseitig)

Im Hinblick auf die geprüften Geschlechtermoderationseffekte gab es lediglich für die Person-Beruf-Kompatibilitätsvariable zum führend-verkaufenden Bereich einen signifikanten Geschlechtergruppenunterschied in der Vorhersage des Weiterverfolgungsentscheids. Weibliche und männliche Jugendliche unterscheiden sich signifikant im Effekt der Person-Beruf-Kompatibilität zwischen führend-verkaufenden Interessen und führend-verkaufenden Anforderungen des Praktikumberuf auf den Weiterverfolgungsentscheid, ∆χ^2^(1) = 6,17, *p* = 0,013. Bei weiblichen Jugendlichen hat die Person-Beruf-Kompatibilität zwischen ihren führend-verkaufenden Interessen und den führend-verkaufenden Anforderungen des ausprobierten Praktikumberufs einen signifikant negativen Einfluss auf den Weiterverfolgungsentscheid (β = −0,17, *p* = 0,022). Ein Überhang führend-verkaufender Anforderungen des Praktikumberufs gegenüber den führend-verkaufenden Interessen führt dazu, dass sich weibliche Jugendliche mit geringerer Wahrscheinlichkeit für die Weiterverfolgung des Praktikumberufs entscheiden. Bei den männlichen Jugendlichen ist dieser Effekt nicht signifikant (β = 0,08, *p* = 0,234).

Da sowohl für das Lehrstellenangebot, ∆χ^2^(1) = 0,82, *p* = 0,365, als auch für die restlichen Person-Beruf-Kompatibilitätsvariablen in den Bereichen handwerklich-technisch, ∆χ^2^(1) = 0,40, *p* = 0,526, untersuchend-forschend, ∆χ^2^(1) = 0,02, *p* = 0,883, künstlerisch-kreativ, ∆χ^2^(1) = 1,92, *p* = 0,166, erziehend-pflegend, ∆χ^2^(1) = 0,06, *p* = 0,812, und ordnend-verwaltend, ∆χ^2^(1) = 0,07, *p* = 0,798, keine signifikanten Geschlechtergruppenunterschiede in der Vorhersage des Weiterverfolgungsentscheids vorgefunden wurden, wurde dieselbe Analyse aus Tab. 1 zusätzlich ohne Geschlechtermoderation gerechnet. Für jene Prädiktoren ohne signifikanten Geschlechtergruppenunterschied in der Vorhersage des Weiterverfolgungsentscheids zeigte sich, dass das Angebot an verfügbaren Stellen einen signifikant positiven Einfluss auf den Weiterverfolgungsentscheid hat (β = 0,25, *p* < 0,001). Je grösser das Lehrstellenangebot für einen von den Jugendlichen ausprobierten Praktikumberuf ist, desto grösser ist die Wahrscheinlichkeit, dass dieser Beruf im Berufswahlprozess weiterverfolgt wird. Hinsichtlich der Person-Beruf-Kompatibilität zwischen den beruflichen Interessen der Jugendlichen und den beruflichen Anforderungen der ausprobierten Praktikaberufe in den Bereichen handwerklich-technisch (β = 0,05, *p* = 0,276), untersuchend-forschend (β = 0,02, *p* = 0,631), künstlerisch-kreativ (β = −0,03, *p* = 0,552), erziehend-pflegend (β = 0,01, *p* = 0,900) und ordnend-verwaltend (β = −0,00, *p* = 0,962) gab es keine signifikanten Effekte auf den Weiterverfolgungsentscheid.

### Post-hoc Analyse

In einem post-hoc Verfahren wurde überprüft, in welchem Ausmaß die führend-verkaufenden Anforderungen eines von den Jugendlichen ausprobierten Praktikumberufs ohne Berücksichtigung der Kompatibilität mit den führend-verkaufenden Interessen einen Einfluss auf den Weiterverfolgungsentscheid haben – unter Kontrolle des Lehrstellenangebots. Ein Regressionsmodell mit den beiden Prädiktoren *führend-verkaufende Anforderungen des Praktikumberufs* und *Lehrstellenangebot zum Praktikumberuf *wurde hierzu gerechnet. Im Modell zeigte sich für das Angebot an verfügbaren Lehrstellen wiederum ein signifikant positiver Effekt auf den Weiterverfolgungsentscheid^1^. Außerdem verwies das Modell auf einen signifikanten Geschlechtergruppenunterschied im Effekt der führend-verkaufenden Anforderungen des von den Jugendlichen ausprobierten Praktikumberufs auf den Weiterverfolgungsentscheid, ∆χ^2^(1) = 5,46, *p* = 0,019. Bei den weiblichen Jugendlichen haben die führend-verkaufenden Anforderungen des ausprobierten Praktikumberufs einen signifikant negativen Effekt (β = −0,23, *p* = 0,002); bei den männlichen Jugendlichen ist der Effekt nicht signifikant (β = 0,04, *p* = 0,632). Bei weiblichen Jugendlichen verringern hohe führend-verkaufende Anforderungen eines Praktikumberufs die Wahrscheinlichkeit eines positiven Entscheids für die Weiterverfolgung dieses Praktikumberufs als potenzieller Ausbildungsberuf im Berufswahlprozess – unabhängig ihrer führend-verkaufenden Interessen und unter Kontrolle des Effekts des Lehrstellenangebots.

## Diskussion

### Zusammenfassung und Interpretation

In diesem Beitrag wurde untersucht, welche Faktoren den Entscheid Jugendlicher für die Weiterverfolgung eines Praktikumberufs als potenzieller Ausbildungsberuf im Berufswahlprozess vorhersagen. Die gerechneten Regressionsmodelle haben gezeigt, dass das Lehrstellenangebot der stärkste Prädiktor bezüglich des Weiterverfolgungsentscheids ist. Dieser Befund entspricht Gottfredsons (2002) TCCSC, wonach Änderungen bei der Berufswahl von Jugendlichen ab dem 14. Lebensjahr hauptsächlich aufgrund einer erschwerten Zugänglichkeit auf sich genommen werden. Mit der TCCSC übereinstimmend und Hypothese 1 bestätigend brachten die gerechneten Modelle hervor, dass sich mit zunehmender Anzahl angebotener Lehrstellen in den von Jugendlichen ausprobierten Praktikaberufen die Wahrscheinlichkeit eines positiven Weiterverfolgungsentscheids erhöht. Im Sinne von Gottfredsons (2002) Theorie findet eine Eingrenzung statt, wobei weniger zugängliche Berufe für Berufe mit höherem Stellenangebot (z. B. Kaufmann/-frau, Detailhandelsfachmann/-frau, Logistiker/-in) aufgegeben werden (Kompromissprozess).

Die Annahme, dass die Wahrscheinlichkeit eines positiven Weiterverfolgungsentscheids umso grösser ist, je grösser die Person-Beruf-Kompatibilität zwischen den beruflichen Interessen der Jugendlichen und den beruflichen Anforderungen der von den Jugendlichen ausprobierten Praktikaberufe ist (Hypothese 2), hat sich ausschließlich für den führend-verkaufenden Bereich bei weiblichen Jugendlichen gezeigt. Hierzu ist jedoch anzumerken, dass die führend-verkaufenden Anforderungen eines Praktikumberufs als berufliche Dimension, d. h. ohne Kompatibilitätsbezug zu den führend-verkaufenden Interessen, den Weiterverfolgungsentscheid der weiblichen Jugendlichen ebenfalls signifikant beeinflussen. Unabhängig von ihren führend-verkaufenden Interessen entscheiden sich weibliche Jugendliche mit größerer Wahrscheinlichkeit gegen die Weiterverfolgung eines Praktikumberufs, wenn dieser Beruf hohe führend-verkaufende Anforderungen stellt. Hypothese 2 wurde somit falsifiziert. Dass bei weiblichen Jugendlichen mit den führend-verkaufenden Anforderungen eines ausprobierten Praktikumberufs auch nach Kontrolle des Lehrstellenangebots eine weitere berufliche Dimension einen Einfluss auf ihre Berufswahl hat, kann auf einen möglichen Kompatibilitätsaspekt hindeuten, welcher in Gottfredsons (2002) TCCSC nur ansatzweise thematisiert wird: die Person-Beruf-Kompatibilität zwischen beruflichen Fähigkeitsselbsteinschätzungen und beruflichen Anforderungen. Möglicherweise fühlen sich weibliche Jugendliche in Praktikaberufen mit hohen führend-verkaufenden Anforderungen hinsichtlich ihrer führend-verkaufenden Fähigkeiten zu unsicher, um diese Berufe in ihrer Berufswahl weiterzuverfolgen. Ihr Selbstkonzept bezüglich der eigenen führend-verkaufenden Fähigkeiten ist mit den führend-verkaufenden Anforderungen eines Berufs nicht kompatibel und es kommt zu einer Abkehr von diesem Beruf als potenzieller Ausbildungsberuf. Weshalb bei weiblichen Jugendlichen von den Anforderungsdimensionen gerade jene im Bereich führend-verkaufend zu einer Abkehr von Berufen führt und welche Rolle berufliche Fähigkeitsselbstkonzepte – besonders zum führend-verkaufenden Bereich – dabei spielen, sollte in zukünftigen Studien weiter untersucht werden. Mit Blick auf andere Berufswahltheorien (z. B. sozial-kognitive Laufbahntheorie von Lent et al. 1994) ist es wahrscheinlich, dass berufliche Fähigkeitsselbsteinschätzungen in einem Kompatibilitätsverständnis den Berufswahlprozess entscheidend mitbeeinflussen.

Im Hinblick auf den vorgefundenen Geschlechterunterschied im Effekt der führend-verkaufenden Anforderungen auf den Weiterverfolgungsentscheid könnten Geschlechterunterschiede in selbsteingeschätzten führend-verkaufenden Fähigkeiten eine Erklärung liefern. Die Forschung zu Geschlechterunterschieden in beruflichen Fähigkeitsselbsteinschätzungen hat gezeigt, dass männliche College-Studierende, aber auch männliche Erwerbstätige, ihre Fähigkeiten im führend-verkaufenden Bereich höher einschätzen als weibliche College-Studierende und Erwerbstätige (Betz et al. 1996).

### Limitationen

Nachfolgend sollen die zentralen Limitationen dieser Studie berichtet werden.

Eine Limitation bezieht sich darauf, dass nicht geprüft wurde, in welchem Ausmaß der Grad der Übereinstimmung zwischen beruflichen Anforderungen und individuellen Fähigkeitsselbsteinschätzungen in diesen Anforderungsbereichen den Weiterverfolgungsentscheid beeinflusst. Der Einbezug dieses Aspekts schafft möglicherweise Klarheit auch im Hinblick auf den negativen Einfluss der führend-verkaufenden Anforderungen eines Berufs auf die Weiterverfolgungsabsichten bei weiblichen Jugendlichen. Im WiSel-Projekt, welches den größten Teil der Daten für diese Studie lieferte, wurden keine Variablen zu beruflichen Fähigkeitsselbsteinschätzungen in den Interessens- und Umweltbereichen nach Holland (1997) erhoben. Zukünftige Studien sollten diesen Aspekt berücksichtigen. Außerdem muss mit Blick auf die abhängige Variable der Studie angemerkt werden, dass ein positiver Entscheid für die Weiterverfolgung eines Praktikumberufs nicht zwingend bedeutet, dass die Jugendlichen den Beruf auch tatsächlich als Ausbildungsberuf nach der obligatorischen Schulzeit erlernten. In der Vergangenheit wurde die Wirksamkeit von Berufswahlpraktika, unter anderem aufgrund der fehlenden individuellen Nachbesprechung der Praktika (eine Folge des mangelhaften Individualisierungsgrads schulischer Berufsorientierung; Driesel-Lange et al. 2013), eher kritisch betrachtet. Neuenschwander et al. (2018) zeigten diesbezüglich jedoch auf, dass zumindest hinsichtlich des Berufsfeldes eine hohe Übereinstimmung zwischen Praktikum- und Ausbildungsberuf besteht. Auch ohne Berücksichtigung des Bewerbungsprozesses kann die Wahrscheinlichkeit als relativ hoch eingeschätzt werden, dass Praktika ihre Wirkung zeigen und Jugendliche mit einem positiven Weiterverfolgungsentscheid denselben Beruf für die berufliche Grundbildung wählen, auch weil die Ausbildungsbetriebe die Praktika häufig als Teil des Selektionsverfahrens nutzen (Schweizerisches Dienstleistungszentrum Berufsbildung 2018). Eine zweite Limitation bezieht sich auf die Variablenkodierungen. Das Lehrstellenangebot wurde mit Daten für die Referenzjahre 2018–2020 erfasst. Die Jugendlichen der verwendeten Stichprobe absolvierten ihre Praktika jedoch größtenteils zwischen 2014 und 2016 und begannen im August 2016 ihre berufliche Grundbildung. Indem das Lehrstellenangebot mittels Daten für verschiedene Jahre gebildet wurde, konnte so zumindest potenziellen jährlichen Schwankungen im Lehrstellenangebot methodisch begegnet werden. Hinsichtlich der Person-Beruf-Kompatibilität zwischen den beruflichen Interessen und den beruflichen Anforderungen der von den Jugendlichen ausprobierten Praktikaberufe muss einschränkend darauf hingewiesen werden, dass die Angaben zu den beruflichen Anforderungen nicht auf Berufsebene, sondern lediglich auf Berufsgattungsebene gemäß detailliertester ISCO-08 Einheit vorlagen. Die Kodierungen konnten entsprechend nicht auf die Praktikaberufe selbst angewendet werden, sondern lediglich auf die zu Berufsgattungen zusammengefassten Praktikaberufen. Dieses Vorgehen führt zu Einschränkungen in der Genauigkeit der Aussagen. Aufgrund der großen Anzahl an Berufen waren Berufskodierungen auf Ebene der Berufe jedoch nicht möglich. Aus demselben Grund wurde das Lehrstellenangebot ebenfalls auf Berufsgattungsebene verwendet. Im Hinblick auf die weiteren Kodierungen der verwendeten Variablen ist anzumerken, dass das Geschlecht der Jugendlichen binär behandelt wurde. Der Einbezug von Geschlecht als mehrkategoriale Variable oder als kontinuierliches Konstrukt ist aus mess- und gendertheoretischer Hinsicht einer binären Behandlung von Geschlecht vorzuziehen (vgl. Döring 2013), war aus methodischen Gründen (z. B. Stichprobengröße) jedoch nicht möglich. Schließlich ist mit Bezug auf die Variablenkodierungen als Limitation festzuhalten, dass die beruflichen Interessen der Jugendlichen im siebten Schuljahr lediglich mit einem Item pro Umwelt- bzw. Interessensbereich (Holland 1997) erfasst wurden. Eine Behandlung der beruflichen Interessen als latentes Konstrukt aus mehreren Items ist der verwendeten Operationalisierung vorzuziehen, da die Interessen dadurch genauer abgebildet werden können.

### Implikationen und Ausblick

Aus den Befunden ergeben sich verschiedene theoretisch-empirische und praktische Implikationen. Auf theoretisch-empirischer Ebene konnte diese Studie die als theoretische Grundlage dienende TCCSC von Gottfredson (2002) in vielerlei Hinsicht voranbringen. Erstens wurden die theoretischen Aussagen mit einer Stichprobe aus Jugendlichen für eine Zielgruppe geprüft, die bislang eher selten untersucht wurde. Zweitens konnte die in der vorliegenden Studie angewendete Methodik gewisse Limitationen früherer Studien überwinden. Die meisten bisherigen Studien zu dieser Thematik verwendeten ein experimentelles Design wie beispielsweise den choice dilemma approach. Bei diesem Verfahren müssen Studienteilnehmende aus mehreren Berufspaaren jeweils einen favorisierten Beruf auswählen. Dem Verfahren wird angelastet, die Realität nur bedingt abzubilden: „Being asked to choose between two occupations paired together by a researcher is very different from a real-life situation in which the individual simultaneously considers a number of occupational alternatives of his or her own choosing“ (Leung und Plake 1990, S. 405). In der vorliegenden Studie wurden konkrete, in der Arbeitswelt ausgeübte Berufswahlhandlungen untersucht, und nicht bloß geäußerte berufliche Präferenzen, die über ein künstliches Studiendesign erhoben wurden. Dass Personen zwischen mehreren beruflichen Optionen abwägen, wurde sowohl bei der Erhebung der Praktikaberufe (Verwendung eines offenen Antwortformats) als auch bei der Auswertung (Berücksichtigung der in Jugendlichen genesteten Berufswahlpraktika) Rechnung getragen. Drittens wurde mit dem Einbezug des Lehrstellenangebots auch der Zugänglichkeitsaspekt berücksichtigt. Gottfredsons (2002) Theorie besagt, dass Personen ab einem gewissen Alter von ihren beruflichen Präferenzen hauptsächlich abkehren, wenn sie Probleme bei der Zugänglichkeit des Berufs wahrnehmen. In bisherigen Studien fehlte dieser altersabhängige Faktor zumeist. Viertens gibt diese Studie Hinweise, welche für die Aufnahme weiterer Kompatibilitätsaspekte zwischen Person und Beruf in Gottfredsons TCCSC sprechen. Die beruflichen Fähigkeitsselbsteinschätzungen als Aspekt des Selbstkonzepts könnten in einem Kompatibilitätsbezug zu den beruflichen Anforderungen für die Vorhersage von Berufsentscheidungen vielversprechend sein.

Neben theoretischen Implikationen ermöglichen die vorgefundenen Ergebnisse die Ableitung konkreter Implikationen für die Praxis. Die Ergebnisse der gerechneten Regressionsmodelle haben gezeigt, dass der Weiterverfolgungsentscheid durch berufliche Dimensionen (Lehrstellenangebot sowie führend-verkaufende Anforderungen bei weiblichen Jugendlichen) bestimmt wird. Vor dem Hintergrund des Befundes, dass in Praktika oftmals angestrebte Berufsfelder erprobt werden (Neuenschwander et al. 2018), stellen die beiden Einflussgrößen in gewisser Weise einen Bruch im individuellen Berufswahlprozess dar: Aufgrund des knappen Lehrstellenangebots und hoher führend-verkaufender Anforderungen (bei weiblichen Jugendlichen) werden Berufe im eigenen Berufswahlprozess nicht weiterverfolgt. Eine Möglichkeit zur Beseitigung der Zäsur wäre eine Vergrößerung der Lehrstellenanzahl in Berufen mit geringem Angebot (z. B. Papiertechnologe/-in, Uhrmacher/-in, Gleisbauer/-in), was jedoch eher schwierig umzusetzen ist. Erfolgsversprechender könnte der zweite Befund zum negativen Einfluss führend-verkaufender Anforderungen auf den Weiterverfolgungsentscheid bei weiblichen Jugendlichen sein. Der Effekt kann als eine wahrgenommene Inkompatibilität aufgefasst werden, wenn weibliche Jugendliche das Gefühl haben, dass ihre führend-verkaufenden Fähigkeiten nicht ausreichen, um hohen führend-verkaufenden Anforderungen in einem Beruf (z. B. Detailhandelsfachfrau/-mann, Drogist/-in, Restaurationsangestellte/-r) gerecht zu werden. Betz et al. (1996) zeigten auf, dass weibliche Erwerbstätige ihre führend-verkaufenden Fähigkeiten tiefer einschätzen als männliche Erwerbstätige. Sollte sich in zukünftigen Studien zeigen, dass bei weiblichen Jugendlichen mit einem hohen führend-verkaufenden Fähigkeitsselbstkonzept der negative Effekt von führend-verkaufenden Anforderungen des gewählten Berufs auf den Weiterverfolgungsentscheid nicht mehr auftaucht, sollten entsprechende Maßnahmen zur Förderung des Selbstkonzepts weiblicher Jugendlichen in diesem Bereich in Betracht gezogen werden. Aus der Forschung zu Fähigkeitsselbsteinschätzungen ist bekannt, dass beispielsweise die Selbstwirksamkeitserwartung von Jugendlichen gesteigert werden kann, wenn sie Aufgaben erfolgreich bewältigen können oder wenn Personen von außen die Jugendlichen in ihren Fähigkeiten bestärken (Betz 1992). Es ist abzuklären, ob weibliche Jugendliche in Praktikaberufen mit hohen führend-verkaufenden Anforderungen ihre in den Praktika zugeteilten Aufgaben selten erfolgreich bewältigen können oder durch Personen der Praktikumsstelle (z. B. Praktikumsverantwortliche) zu wenig in ihren führend-verkaufenden Fähigkeiten bestärkt werden.

Neben der Klärung dieser Frage gibt es weitere wichtige Forschungsdesiderata, die sich aus dieser Studie ableiten lassen. Erstens sollten zukünftige Studien den weiteren Berufswahlprozess nach dem Absolvieren von Praktika erforschen. Die Frage, in welche Ausbildungsberufe weibliche Jugendliche nach dem Ausprobieren von Praktikaberufen mit hohen führend-verkaufenden Anforderungen wechseln, stellt ein wichtiges Forschungsdesiderat dar. Ebenso sollte überprüft werden, welche Eigenschaften jene Ausbildungsberufe haben, in welche Jugendliche wechseln, die ihre Praktika in Berufen mit tiefen Lehrstellenangebot absolvierten. Die Bearbeitung dieser Fragen kann zu einem besseren Verständnis des Berufswahlprozesses von Jugendlichen beitragen. Zweitens wäre es aus forschungsmethodischer Hinsicht interessant, mittels *random intercept and random slope* Modellen zu untersuchen, welche Rolle die Reihenfolge der absolvierten Praktika für den Weiterverfolgungsentscheid spielt. Entscheidungsprozesse ließen sich so noch genauer nachzeichnen. Drittens könnten zukünftige Studien untersuchen, welcher Zusammenhang zwischen den mit einem Praktikumberuf verbundenen beruflichen Dimensionen und den in den Praktika gemachten Erfahrungen (z. B. Qualität des Praktikums, wahrgenommene Arbeitsbelastung, Zufriedenheit mit dem Beruf etc.) besteht und wie die konkreten gemachten Erfahrungen den Weiterverfolgungsentscheid beeinflussen. Bei Vorliegen negativer Erfahrungen könnten mögliche Maßnahmen eruiert werden, um die Situation in den Praktika zu verbessern. Ein viertes Desiderat bezieht sich auf die Kodierung der zu den Praktikaberufen zugehörigen beruflichen Dimensionen. In dieser Studie wurden die beruflichen Dimensionen über „objektive“ Kriterien kodiert. Vergangene Studien lieferten Hinweise, dass sich eine auf subjektiven Einschätzungen basierende Kodierung von einer auf objektiven Kriterien basierenden Kodierung wesentlich unterscheidet (Leung und Plake 1990). Inwiefern sich die in dieser Studie vorgefundenen Befunde auch bei einer auf subjektiven Einschätzungen basierenden Kodierung zeigen, ist empirisch zu überprüfen. Die Bearbeitung all dieser Fragen kann wichtige Erkenntnisse zum ersten Berufswahlprozess von Jugendlichen liefern und an den wegweisenden Befunden dieser Studie anknüpfen.

## Footnotes

1 Für weibliche Jugendliche: β = 0,31, *p* < 0,001, für männliche Jugendliche, β = 0,14, *p* = 0,074, wobei der Geschlechtergruppenunterschied im Effekt nicht signifikant ist, ∆χ^2^(1) = 1,31, *p* = 0,252. Geschlechtsunspezifischer Wert: β = 0,26, *p* < 0,001.
